# Teacher Technostress in the Chilean School System

**DOI:** 10.3390/ijerph17155280

**Published:** 2020-07-22

**Authors:** Carla Estrada-Muñoz, Dante Castillo, Alejandro Vega-Muñoz, Joan Boada-Grau

**Affiliations:** 1 Departamento de Ergonomía, Universidad de Concepción, Concepción 4070386, Chile; carlaestrada@udec.cl; 2 Centro de Estudios e Investigación Enzo Faletto, Universidad de Santiago de Chile, Santiago 9170022, Chile; dante.castillo@usach.cl; 3 Facultad de Administración y Negocios, Universidad Autónoma de Chile, Providencia 7500912, Chile; 4 Departamento de Psicología, Universidad Rovira i Virgili, 43007 Tarragona, Spain; joan.boada@urv.cat

**Keywords:** anxiety, confirmatory factor analysis, education, fatigue, inefficacy, information overload, principal components analysis, skepticism

## Abstract

The expanded use of information technology in education has led to the emergence of technostress due to a lack of adaptation to the technological environment. The purpose of this study is to identify the levels of technostress in primary and secondary education in 428 teachers using a RED-TIC questionnaire, of which skepticism, fatigue, anxiety, and inefficiency are the main components. For the empirical analysis of the data, principal component analysis (PCA) and confirmatory factor analysis (CFA) were used. The results show that 12% of Chilean teachers participating in the study feel techno-fatigued, 13% feel techno-anxious, and 11% present both conditions. Male teachers show a higher incidence of techno-anxiety and techno-fatigue than their female peers. It can be concluded that the questionnaire used is a reliable tool to evaluate the presence of technostress, and it manifests itself importantly in its components of techno-anxiety and techno-fatigue in Chilean teachers.

## 1. Introduction

Psychic and mental conditions significantly influence people’s overall health, and particularly their daily performance [1,2,3,4,5]. According to the latest annual statistical report of the Chilean Social Security Superintendence, mental health disorders are the main cause of medical licenses (necessary in order to be absent or reduce one’s work day during a certain period of time) in 20% of the total number of authorized medical licenses. Of these licenses by mental health disorders, 76% correspond to females, in case of the public health system (FONASA), and 59% correspond to females, in case of the private health system (ISAPRE) [6].

This relationship between health and performance is seen in labor relations, especially in professions that are linked daily to communities, beneficiaries, or clients. In this way, this is particularly important for teachers in the school system, because they are connected and have a responsibility to educate children and young people. The relationship between teachers and students, given the social relevance and the prolonged time during which the two interact, exerts a decisive influence on students’ lives. It is therefore relevant to analyze the mental health and psychosocial risk manifestations of teachers, for both the well-being of themselves as well as for the effects it has on the teaching process and on students’ development.

Work stress manifestations and teachers’ mental health have been a topic of interest for at least two decades; in Anglo-Saxon and European societies, mental health concern has been perceived since the early 1970s, while in Chilean society, more systematic efforts have been made to delve into this topic since the 1990s [7]. The unexpected relevance of concern for teachers’ mental health has a double explanation. On the one hand, since the educational reforms of the 1990s, expert discourse has argued that Chilean education has had significant deficiencies related to the low motivation and low participation of teachers in curriculum changes as well as a lack of adaptation to social changes, explained by the mental attitude of teachers [8,9,10].

On the other hand, concern for teaching health has also been explained by studies carried out by the United Nations Educational, Scientific, and Cultural Organization’s (UNESCO) Regional Office for Latin America and the Caribbean (OREALC). The most significant being the Regional Comparative and Explanatory Studies, which periodically seek to assess the learning achievements of Latin American students. In some of these, educational success factors associated with the psychosocial condition of teachers were identified [11,12]. However, interest was concentrated on the environmental situations of the educational institution, with a focus on the climate and on school coexistence, overshadowing the personal condition of teachers. In other words, work stress in teachers was overshadowed by corporate health.

In this context, work stress assessment or work characteristics reviews carried out by teachers were postponed. Teachers’ professional attributes go beyond their working hours and daily tasks. Thus, in the case of teachers, analyzing work stress by only considering the elements of work done in the classroom work would neglect the emotional and moral burden of teaching. In short, teachers work every day and at all times. This is because of the peculiar characteristics of the teaching profession, which can potentially cause significant stress and psychosocial damage [13]. As Golembiewski et al. showed [14], worldwide, the teaching profession is strongly related to higher stress levels. Teaching seems to have an inherent component of potentiality for stress, fatigue, and states of inner unease. In this way, at an international level, there has been strong interest in undertaking studies on work stress in teachers. In terms of gender distinction, in 2007, Oramas et al. [15] reported on a study conducted between 1997 and 1999 by Weber et al. (2015), in which all 408 cases of early retirement in teachers were reviewed. In that study, 45% gave psychosomatic and psychiatric disorders as a cause, with a higher incidence occurring in women than in men, which is historically due to the double presence or work–family conflict among women [16,17]. This was the main gender difference identified between psychosocial risks in Chile after the SUSESO/ISTAS21 questionnaire in 2016, with an odds ratio (female/male) of 1.59, which decreased between 2017 and 2019, with values of 1.59, 1.09, and 1.02, respectively [18,19,20,21,22]. These psychiatric disorders included depression and emotional exhaustion. In another study by Lodolo-D’Oria et al. [23], four professions were compared, namely teachers, office workers, health professionals, and utility workers, in relation to mental disorders, and it was concluded that the risk of developing psychiatric disorders for teachers is 2 times, 2.5 times, and 3 times greater than for office workers, health professionals, and utility workers, respectively; once again with a higher incidence in the case of female teachers. However, although interest in work stress in teachers in Chile has significantly declined since the new millennium, the expansion and use of computer and communication technology in Chilean schools has offered a new opportunity to investigate the psychosocial and mental risk manifestations associated with these new resources and methodologies.

This article describes a research project performed in the second half of 2019, which deals with the relationship between the incorporation and mass use of computer and communications technology in teaching and learning. The research took place in primary and secondary schools in the surrounding regions of Valparaiso and the Metropolitan of Santiago, Chile.

The purpose of this publication is to approximate the state of the mental health of Chilean teachers who work in public and private schools with a state grant, in relation to the incorporation of computer and communication technology. To this end, we set out to use the criteria used by the Spanish Ministry of Labor, after validation in Chile, to diagnose some technostress manifestations associated with the mental health of education workers.

## 2. Background

The use of technology can lead to beneficial transformative changes within an organization; however, it can also lead to negative consequences in job satisfaction deterioration, commitment, work continuity, productivity, and morale, as well as increased work overload and work–life conflict [24,25,26]. These negative effects are further emphasized with the ubiquity of mobile devices connected to the network and through continued work development, even after working hours [25]. In this regard, O’Driscoll et al., cited by Day et al. [27], point out that the approach of increasing employees’ accessibility to their “work environment” and increasing their productivity expectations through the use of information technology increases workload requirements. Thus, the use of information technology creates an imbalance between the demands and control resources of users, overcoming the possibility of being able to self-manage stressors [28,29,30,31].

The concept of technostress was first noted in mainstream magazines in 1982 by Craig Brod, as a condition resulting from an individual and/or organizational inability to healthily adapt to new technology use, which is modulated according to age, previous techno experiences, workload, perception of control, and working climate, and consequently affects people’s performance, thus limiting their use of technology [32,33]. In general, the concept includes the adverse effects caused by technology on people’s attitudes, thoughts, behaviors, and physiology [34]. In this regard, as psychosomatic consequences are recognized, namely, sleep problems, headaches, muscle aches, and gastrointestinal disorders, in the long term, teachers may end up developing exhaustion (burn-out syndrome) [29]. People who experience psychological and emotional rejection to information technology, for example, experiencing breakdowns, fear, tension, or anxiety, may stop or prevent their ability to learn [35].

Technostress develops from the concurrence of multiple and intense stress conditions experienced by the worker in the extended organizational environment, whose dynamics promote tension, known as techno-stressors, which can be present in any work environment where computers are used. These technological stressors are technological invasion, technological overload, technological complexity, technological insecurity, and technological uncertainty [26,35,36,37]. In accordance with the ideas of Ayyagari et al. [38], stressors can be caused by tasks (work overload, work schedule, and exposure to risks and dangers), role characteristics (ambiguity, conflict, and overload), interactions within the organization (interpersonal relationships and leadership style), career (work insecurity and career advancement), organizational factors (climate and structure), work–home interface (work–home conflict and privacy invasion), and characteristics related to the physical work environment (noise, temperature, and vibration), all of which can be accentuated with the use of information technology at work.

It should be added that techno-stressors do not necessarily have a direct effect, and may be mediated by employees’ stress or fear and through their coping strategies, consistent with the stress dynamics and coping theory of Lazarus [39,40]. Additionally, technostress unfolds among related constructs such as “information fatigue syndrome” or techno-fatigue, techno-addiction, and technophobia [41]. From another perspective, the organization of the internal environment influences technostress levels in workers; in the data, a significant positive relationship can be found between technostress levels and a centralized power structure and an organizational environment oriented to innovation [35].

High levels of stress can affect people, even having direct negative effects on health. For example, subjects exposed to the repeated malfunction of information technology, namely collapsing computer systems, showed increased levels of cortisol (a stress-associated hormone), with their average levels sharply increasing directly after a system collapse, which could affect a person’s health [34]. From a management perspective, there must be a situation of balance between people and their environment, so that there is no tension state [38,39,40,41,42]. Therefore, companies can facilitate adaptation strategies by improving the internal knowledge of their information systems; reducing stressful technological factors of work environments by reducing the exhaustion of their workers; and, in general, considering the interaction between techno-stressors, technostress, and coping strategies [25,26].

In the education sector, technostress has been studied in the last two decades, with various focuses; on the one hand, employees in the educational system have been selected in order to access certain groups of the population [43,44]. On the other hand, educational processes have been directly considered; studies on technostress have been identified in library processes [45,46] and there are other publications that advise about technostress among university students and their learning processes [47,48,49,50], computer literacy, and digital thinking [51], as well as with their use of digital textbooks [52]. However, the study group of interest for this research is that related to the teaching role [53,54,55,56,57,58,59].

The initial literature regarding teacher technostress considers it to be caused by the introduction of technology into the classroom and from a lack of adaptation to the technological environment [53]. This can be reduced when teachers receive administrative support for the use of technology (continuous access to technical support and updated technology for the preparation and development of their activities), which gives them a supporting atmosphere [54]. That support influences technostress, which, in turn, affects the intention of the technological use by teachers [56]. Recent research on Indian and Chinese higher education emphasizes the presence of techno-stressors and techno-inhibitors that influence job satisfaction; organizational commitment; negative affectivity due to work; and, above all, technology-mediated performance [55,57,58,59]. In general, the lack of adaptation between people and their work environment affects their job performance [57]. Studies that focus on South Korean and Chinese education also incorporate a model based on teaching measurement based technology, pedagogy, and content knowledge (TPACK) as a study variable [56,57,58,60].

Thus, this study focuses on measuring the psychological state related to the use of information technology in primary and secondary school teachers in order to identify manifestations of the psychosocial risks, contributing to expanding on studies at a school level and reporting on the first empirical study in Latin America.

**Figure 1:**
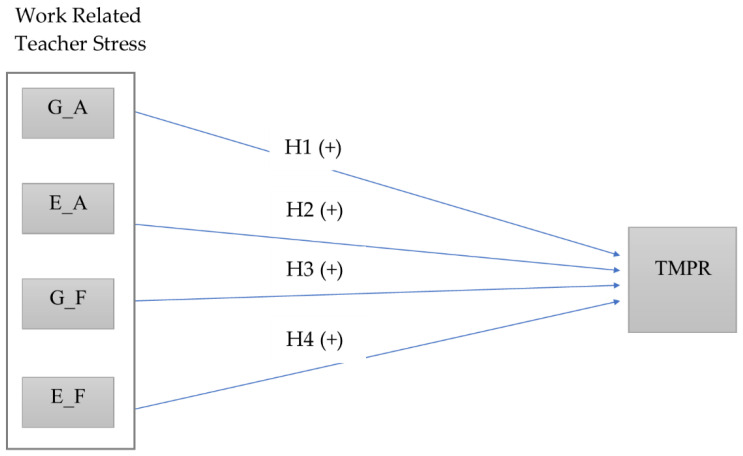
Research model and hypotheses. Notes: GA, faculty gender in techno-anxiety manifestation; EA, faculty age section in techno-anxiety manifestation; GF, faculty gender in techno-fatigue manifestation; EF, faculty age section of teachers in techno-anxiety manifestation; TMPR, technostress manifestations in psychosocial risk. Source: own elaboration.

According to the above literature, the following research hypothesis were set out for this article (Figure 1):

**Hypothesis** **1** **(H1).** *There is a statistically positive relationship between the gender of teachers and the techno-anxiety levels measured by a technostress instrument*.

**Hypothesis** **2** **(H2).** *There is a statistically positive relationship between the age groups of teachers and the techno-anxiety levels measured by a technostress instrument.*

**Hypothesis** **3** **(H3).** *There is a statistically positive relationship between the gender of teachers and the techno-fatigue levels measured by a technostress instrument.*

**Hypothesis** **4** **(H4).** *There is a statistically positive relationship between the age groups of teachers and the techno-fatigue levels measured by a technostress instrument.*

## 3. Methods 

### 3.1. Participants

The database of teachers was obtained through probabilistic sampling. The sample was stratified into three groups depending on the type of educational center, namely, public, private with state grants, and private without state grants. Tiered sampling allowed for reducing the variation in results due to the strata of the population and for obtaining a greater accuracy in the estimates [61,62]. The optimal sample size was calculated using a statistical procedure in order to reject a null hypothesis, when in fact that hypothesis is false or has the potential to avoid a type II error.

The sample size calculation was performed considering a maximum acceptable error of 5%, a confidence level of 95%, and a 50% variance assumption. The population consisted of 105,970 teachers, corresponding to the regions of Santiago and Valparaiso (35,804 teachers from public schools, 53,437 teachers from private schools with state grants, and 16,729 teachers from private schools without state grants) [63]. Under these parameters, the total sample was 428 teachers working in primary and secondary schools (152 public school teachers, 210 state-subsidized private school teachers, and 66 unsubsidized private school teachers).

In relation to the teacher sample characteristics, 276 were women (64.5%) and 152 men (35.5%). The age ranged from 23 to 67 years old, with an arithmetic average of 39.6 years. In addition, 262 teachers were employed with an indefinite contract (61.2%), while 160 teachers had a fixed-term contract (37.4%). At the same time, 392 teachers had a professional degree (91.6%), and 36 teachers taught in schools without having a professional degree (8.4%).

### 3.2. Procedure

The RED-TIC questionnaire integrated into the Technical Note of Prevention 730 of the National Institute for Safety and Hygiene at Work of Spain, which focuses on intra-labor psychosocial risks as a product of the techno-demands, and on a lack of techno-resources and personal resources [29,64], was used as a basis. This questionnaire is composed of skepticism, fatigue, anxiety, and inefficiency dimensions (see Appendix A). This is reliable for the teaching function in Chile, with a Cronbach’s Alpha 0.941 and a Cronbach’s Alpha based on standardized items of 0.946 [31]. For the empirical analysis of the data, the principal component analysis (PCA) was used, a type of multivariate statistical analysis previously used in teaching-stress research [65,66,67,68,69]. Along with this process, the consistency of dimensions was analyzed using Cronbach’s Alpha, as presented in previous research [29]. PCA allows for reducing the dimensionality of the data to the principal components based on statistical and sociological criteria [70].

Additionally, Bartlett’s sphericity test was performed in order to assess the relevance of PCA under the hypothesis of multivariate normality, and the Kaiser–Meyer–Olkin (KMO) test was used as the factorial analysis in order to establish the feasibility of the obtained data [70,71].

### 3.3. Data Analysis

For the data analysis, R statistical analysis software was employed using the RStudio interface, and the sorting and descriptive exploration processes of the data were carried out with the metapackage “Tidyverse” [72] and with “Summarytools” [73]. The principal component analysis was performed with the “FactoMineR” and “FactoExtra” packages [74,75], and the Bartlett and KMO sphericity tests were performed using the “REdaS” package [76]. For the factor rotation, the “psych” package [77] was used and the “Varimax” method was employed. The confirmatory factor analysis was performed with the CFA function of the “lavaan” package [78].

## 4. Results

### 4.1. Principal Component Analysis

The results were obtained using the principal component analysis (PCA) for the 16 variables or reagents included in the technostress instrument, in order to empirically estimate the reliability of the instrument. In this way, the data showed a good correlation, while the Bartlett’s sphericity test indicated an appropriate value (χ^2^ = 4520.424; df = 120; *p* < 0.01); thus, $H_{0}$ was rejected and it was assumed that there were differences between the observed correlation matrix and the identity matrix. The KMO index was 0.91, indicating a very good or optimal value for continuing the factor analysis.

The extraction of components using the analysis of the initial self-value indicated that three dimensions were detected with values over 1. In the classic recommendation for the selection of the number of factors, the Kaiser rule suggests choosing all auto values greater than 1. The component matrix gave factorial loads ≥0.40; the first dimension concentrated on factorial loads of a higher value, except for the r_1 and r_2 variables, which correlated with the second extracted dimension, and the third dimension acquired a greater correlation with the r_5 variable (−0.54). However, the same variable had a larger load with one dimension (0.56). In the rotated solution, the loads could be observed in a different distribution, where rotated component 1 (RC1) mostly contained the loads of the variables r_5 and r_9 to r_16; RC3 had r_1, r_2, r_6, r_7, and r_8 loads; and RC2 had r_3 and r_4 loads. Additionally, the Cronbach’s Alpha generated an alpha value of 0.92 and a standardized alpha of 0.93. The minimum alpha value of a variable was 0.91.

### 4.2. Confirmatory Analysis Factorial

To validate the instrument globally, a confirmatory factor analysis was performed (CFA) [79]. All of the items turned out to be significant, and the goodness of fit indexes met the established criteria, namely: the comparative adjustment index (CFI) was 0.90 [80], the root mean square error of approximation (RMSEA) was 0.103 [81], the Tucker Lewis Index (TLI) was 0.90 [82], and the residual standardized root mean square (SRMR) was 0.05 [83]. To perform the validation, the CFA function of the lavaan package was used [78].

### 4.3. Tecnostress in Chilean Teachers

The technostress instrument was built from four subscales, which, when composed, allowed for identifying two types of technostress manifestations because of the presence of intra-labor psychosocial risks. Thus, high scores in those dimensions will be technostress indicators in its two manifestations: (1) techno-anxiety (high scores in anxiety, skepticism, and inefficiency) and (2) techno-fatigue (high scores in fatigue, skepticism, and inefficiency). However, with the reliability provided by the evaluation instrument, the results showed that, in the case of Chilean teachers, 11.9% were techno-fatigued, and another 13.1% showed a techno-anxious status. Furthermore, 10.7% of this population presented both pathologies (Table 1). In other words, in a school of 50 teachers, at least five of them should be on occupational sickness medical leave.

### 4.4. Technoanxiety Manifestations in Chilean Teachers

Techno-anxiety, as a work pathology, is the best-known type of technostress, where a person experiences high levels of non-pleasant physiological activation and feels tension and discomfort from the present or future use of some type of information and communication technology (ICT). The same anxiety leads to skeptical attitudes about the use of technology, as well as negative thoughts about one’s ability and competence in the use of information technology. A specific type of techno-anxiety is technophobia, which focuses on the affective dimension of fear and anxiety towards ICT. However, to guide public policies, data were analyzed based on the variable of gender. In this regard, it was interesting to note that male teachers were more techno-anxious than their female peers (Table 2).

To confirm the trends observed between the two subpopulations, Student’s t-test confirmed the presence of statistically significant differences between the two groups of teachers (Table 3). In other words, for the Chilean case, the processed information confirmed, in terms of gender, that male teachers showed a higher incidence of techno-anxiety than their female peers. 

Regarding the second hypothesis (H2), which is concerned with a statistically positive relationship between teaching staff age groups and techno-anxiety levels, the results did not show a statistically significant association between both variables. This is confirmed by the value shown by the Pearson’s R bilateral correlation test (Table 4).

### 4.5. Techno-Fatigue Manifestations in Chilean Teachers

Moreover, techno-fatigue is characterized by feelings of exhaustion and mental and cognitive exhaustion due to the use of technology, which is also accompanied by skeptical attitudes and inefficiency beliefs regarding the use of information technology. A specific type of techno-fatigue is the so-called “information fatigue” syndrome, derived from the current requirements of the information society; this is caused by information overload when the Internet is used. Symptomatology is a lack of competence to organize and assimilate new information derived from the use of the Internet, with the consequent appearance of mental fatigue. Based on the obtained data, it could be also established that this pathology tends to be more present in male teachers (Table 5).

The tendency has been supported by the Student’s t-test, which indicates the presence of statistically significant differences between male teachers and their female peers (Table 6).

Regarding the fourth hypothesis (H4), which proposes a statistically positive relationship between teachers’ age groups and the techno-fatigue levels measured by the technostress instrument, the results of the R Pearson test show that there was no statistically significant correlation between both variables, as indicated in Table 7.

## 5. Discussion

The changes generated by new technology require studies so as to avoid risks and negative effects on schools and teachers. Additionally, a history of prevention regulations and prevention services, assessing risks, and identifying working conditions that may be affected by the intensive introduction of the so-called new technology is needed. As a result, using empirical backgrounds, we need to address the effects of technological innovations on education in order to prevent negative impacts and enhance positive ones, both individually and organizationally.

To date, several psychosocial research groups have studied the various expressions and consequences of the introduction of information and communication technology (ICT) into people’s health at work, such as muscle problems, headaches, mental and physical fatigue, anxiety, and fear. However, considering the intensive use currently given to telework, the term technostress has become more important. It should be understood as a manifestation of psychosocial risk specifically related to stress derived from the introduction and use of new technology at work.

For the first time, based on the criteria used by the Spanish Ministry of Labor and Social Affairs through the National Institute of Safety and Hygiene at Work, previously adapted to the Chilean context, this study considered technostress manifestations in the teaching population. This provided up-to-date empirical evidence and enabled the exploration of the relationships between the use of technology in teaching work and technostress levels. This study has become especially relevant because in the context of the global health crisis resulting from the Covid-19 pandemic, public and private institutionalism is drastically driving the incorporation of computer and communications technology in all areas of work activity; teaching and educational work are concentrating almost exclusively on technological media. 

### 5.1. Theoretical Implications

From the analysis of the results based on the proposed hypothesis models, it was expected that age could influence the techno-anxiety or techno-fatigue manifestations observed in teachers. This was supported by general theories of aging. In other words, to develop our H2 and H4 hypotheses, it was proposed that there was enough background to point to a relationship between age and technostress. On the one hand, aging is connected to physical degeneration processes such as cognitive decline [84,85], which make older adults more likely to be exposed to some techno-stressors [86]. On the other hand, aging also seems to be connected to a greater recovery. Thus, older age groups would have a broader collection of strategies to more efficiently address the management of emotions [87,88]. Based on the above, our assumptions suggested that such age-related gains led to a more efficient confrontation with techno-stimulating factors. As a result, age would reduce the tension associated with the use of technology in teaching. Despite this, the obtained results contradicted—at least from the work activity of teachers—the theoretical background, and a significant relationship between age and techno-anxiety or age and techno-fatigue could not be demonstrated. Finally, a high correlation and significance was observed between the variables of age and professional teaching experience in years (Pearson’s R correlation: 0.924, with a bilateral significance of 0.000); thus, the trends between age and years of teaching experience are equivalent.

Regarding the relationship between gender condition and job stress, it is important to consider that workplace demands will establish more pressure on the female gender (as the individual maintains its link relationship from the social role that is assigned), so the “erudite discourse” considers that gender is a stress experience moderator. This would be explained by the associated roles and behavioral expectations of different genders. In this sense, some studies have pointed to two conflicting results related to the experience of stress and gender. On the one hand, some evidence demonstrates that there are no differences between men and women [89], while on the other hand, there is also evidence that gender can cause significant differences. Some studies have found that men are significantly more affected by stress [90,91,92]; however, at the opposite end, other studies have indicated that women are the most affected by stress [93,94,95,96].

When gender is related to stress, the differences between men and women are more reflected in the elements that cause stress and in their coping mechanisms. In this sense, studies that have determined gender differences argue that, for men, the elements that cause work stress are a lack of control over working conditions, and achievement and possibilities for career development; whereas in the case of women, causes of stress appear from being in a high position within the hierarchical structure, and the relationship established between domestic and employment responsibilities [91,97,98,99]. Despite this, from the results provided in this study and as pointed out by the above-mentioned studies, there is significant statistical evidence showing the relationship between gender and technostress manifestations. This is why, in both techno-anxiety and techno-fatigue, male teachers showed higher levels of stress than their female peers. In this way, the H1 and H3 proposals of the hypotheses used in this research are accepted. 

### 5.2. Practical Implications

This study took place in a scenario that we call “normal work”, showing that 1 in 10 Chilean teachers present with a pathology linked to the use of technology. Therefore, it is perfectly possible to infer, in a scenario of social distancing and confinement, where the use of technology is promoted as a mediating channel, that the different manifestations of technostress could be significantly increased.

This article has focused on contributing to the scientific literature on the psychological state of teachers because of their interaction with information and communication technology, and their use as mediators of the teaching profession. It also allows for evaluating the implications that the use of information technology have on the health and well-being of education workers, incorporating a gender and age perspective.

The incorporation of the Internet, mobile telephony, telecommuting, and other resources that the information society is introducing into schools is changing not only the pedagogical processes; all these technological changes are also expressed in the appearance of new occupational pathologies. Unfortunately, this has been scarcely analyzed in Latin American societies. These changes highlight technical problems, but also human and social problems. Therefore, it is imperative to start an in-depth debate, especially because of the consequences for teachers, students, and families who are directly and indirectly affected.

This investigation recognizes the need to prevent risks and avoid negative effects on teachers and schools. It contributes to visualizing how the technostress effects of teaching need to result in rethinking educational policies, considering the incorporation of the digital world into the initial teacher training and professional development [100], the articulation of the recent telework law [101] with the labor regime exclusive to the “teaching statute” [102], and to what extent the principles of the General Education Law may be affected (universality and permanent education, free, quality of education, equity, autonomy, diversity, responsibility, participation, flexibility, transparency, integration and inclusion, sustainability, interculturality, and dignity of the human being, integral education) [103].

### 5.3. Limitations and Future Research

This article generates the need to explore the effects of the massive technological innovations that have been introduced in Chilean schools. The findings of this first study show the need to prevent negative impacts and promote positive ones, both individually and organizationally. In this way, this psychosocial research opens a systematic research line on the consequences of the introduction of information technology on teachers’ mental health and their professional performances in Chilean schools.

Regarding Chile and its mental health in 2019, 24.3% of medical diagnoses were associated with this cause, with an increase of approximately 15% being observed in the first four months of 2020, and with medical diagnoses related to mental disorders accounting for 29% of the total diagnoses for this year [104]. The relationship with the Chilean spring [105], prolonged confinement, teleworking, and information overload are still unknown aspects in education, as well as in other economic sectors, thus encouraging further research.

Regarding this study’s limitations, it is important to consider that this research was conducted in only two regions of the country. Therefore, in future studies, it will be important to refine the sample design and increase the size in order to have national representation. Alongside the above, it is important to improve the hypothesis model by incorporating other factors associated with technostress manifestations, especially those related to different types of teachers working in school institutions. Finally, RED-TIC focuses on intra-labor psychosocial risks associated with the use of technology, without considering the influence of possible extra-work risks and those related to someone’s personal or individual life [106].

## 6. Conclusions

The objective of this study was measuring the psychological state of teachers who work in primary and secondary schools, with regards to the effect of information and communication technology. Altogether, this study attempted to identify the manifestations of psychosocial risks. The results showed the methodological relevance of measuring stress manifestations in education professionals. This statement is supported by the results of the reliability analysis conducted by the questionnaire; a relevant fact that allows for it to be used for deeper research fulfillment and at a national level.

Along with the above, the analysis of the results determined that 13% of the total teachers presented a techno-anxiety condition, while 12% experienced techno-fatigued conditions. In other words, the use of technology is showing its dark side, with adverse effects and psychosocial risks in a significant subset of Chilean teachers. Moreover, the data showed that more than 10% of teachers jointly state both techno-anxiety and techno-fatigued psychosocial manifestations. That is, at least 1 out of 10 Chilean teachers is at psychosocial risk because of the relationship established with the use of information and communication technology. This is expressed with more intensity in the case of male teachers.

From these results, it can be inferred that, together with having effects on teachers’ work, there must also be negative effects on the teaching and learning process. In this regard, there is strong evidence that demonstrates the relationship between teachers’ mental health and the effect on educational relationships, especially in the classroom.

Therefore, this study opens up the debate about the education sciences on the theoretical–practice analysis of technology-use implications in the pedagogy sector, where its appropriate use could have negative consequences for teachers as well as students. This study also guides the generation of specific lines of research that go in depth on the impact of technology use in different fields of scholar culture. Thus, future analyses should focus on the relationships that might be occurring between different teacher profiles and the negative manifestations of technology use.

## Figures and Tables

**Table 1:** Cross between techno-fatigue and techno-anxiety.

Technostress Manifestations		Techno-Anxiety		Total
		No	Yes	
Techno-fatigue	No	85.7%	2.3%	88.1%
	Yes	1.2%	10.7%	11.9%
Total		86.9%	13.1%	100.0%

**Table 2:** Techno-anxiety statistics by gender.

Technostress Manifestation	Sex/Gender	N	Mean	Standard Deviation	Standard Error of the Mean
Techno-anxiety	Masculine	152	6.6546	1.99044	0.16145
	Female	276	5.8505	1.79459	0.10802

**Table 3:** Techno-anxiety Student’s t-test.

Student’s t-Test	Levene’s Test Equality Variance		T-Test for Equality of Means						
Techno-Anxiety Index	F	Sig.	t	df	Sig. (Two-Tailed)	Mean Diff.	Std Error Diff.	95% Conf. Interval Diff.	
								Lower	Upper
Equal variance assumed	4.210	0.041	4.265	426	0.000	0.80406	0.18851	0.43353	1.17459
Equal variance not assumed			4.139	285.08	0.000	0.80406	0.19425	0.42171	1.18641

**Table 4:** Bilateral correlation between teaching age and techno-anxiety.

Bilateral Correlation		Teaching Age	Techno-Anxiety Scale
Teaching age	R Pearson correlation	1	0.022
	Sig. (bilateral)		0.652
	N	428	428
Techno-anxiety scale	R Pearson correlation	0.022	1
	Sig. (bilateral)	0.652	
	N	428	428

**Table 5:** Techno-fatigue statistics by gender.

Technostress Manifestation	Sex/Gender	N	Mean	Standard Deviation	Standard Error of the Mean
Techno-fatigue index	Masculine	152	6.02	2.502	0.203
	Female	276	5.24	2.244	0.135

**Table 6:** Techno-fatigue Student’s t-test.

Student’s t-Test	Levene’s Test Equality Variance		T-Test for Equality of Means						
Techno-Fatigue Index	F	Sig.	t	df	Sig. (Two-Tailed)	Mean Diff.	Std Error Diff.	95% Conf. Interval Diff.	
								Lower	Upper
Equal variance assumed	4.599	0.033	3.298	426	0.001	0.779	0.236	0.315	1.243
Equal variance not assumed			3.196	283.85	0.002	0.779	0.244	0.299	1.259

**Table 7:** Bilateral correlation between teaching age and techno-fatigue.

Bilateral Correlation		Teaching Age	Techno-Fatigue Scale
Teaching age	R Pearson correlation	1	−0.017
	Sig. (bilateral)		0.724
	N	428	428
Techno-fatigue scale	R Pearson correlation	−0.017	1
	Sig. (bilateral)	0.724	
	N	428	428

## Appendix A

In this appendix the four variable sets RED-TIC and their rating scale from 0 to 6 are presented.

**Skepticism set**

S1. With time passing, ICT interests me less and less

S2. Every time I feel less involved in the use of ICT

S3. I am more skeptical about the technology’s contribution to my work

S4. I doubt the working meaning with this technology

**Fatigue set**

F1. I find it difficult to relax after a workday using ICT

F2. When I finish working with ICT, I feel exhausted

F3. I am so tired when I work with ICT that I cannot do anything else

F4. It is hard to concentrate after working with ICT

**Anxiety set**

A1. I feel tense and anxious when working with ICT

A2. It scares me to think that I can destroy a lot of information with the improper use of ICT

A3. I hesitate using ICT for fear of making mistakes

A4. Working with ICT makes me feel uncomfortable, irritable, and impatient

**Inefficacy set**

I1. In my opinion, I am inefficient at using ICT

I2. It is difficult to work with ICT

I3. People say that I am inefficient at using ICT

I4. I am unsure of finishing my tasks well when I use ICT

**Perceptual Rating Scale:**

0. Nothing/never

1. Almost nothing/a couple of times a year

2. Rarely/once a month

3. Sometimes/a couple of times a month

4. Enough/once a week

5. Often/a couple of times a week

6. Always/every day

## References

[1] Dixon L., Goldberg R., Lehman A., McNary S. (2001). The impact of health status on work, symptoms, and functional outcomes in severe mental illness. J. Nerv. Ment. Dis..

[2] Berardi D., Berti-Ceroni G., Leggieri G., Ricci P., Ustun B., Ferrari G. (1999). Mental, physical and functional status in primary care attenders. Int. J. Psychiatry Med..

[3] Beer J., Beer J. (1992). Burnout and stress, depression and self-esteem of teachers. Psychol. Rep..

[4] Wells K., Stewart A., Hays R., Burnam A., Rogers W., Daniels M., Berry S., Greenfield S., Ware J. (1989). The functioning and well being of depressed patients: Results from the medical outcomes study. JAMA.

[5] Broadhead W., Blazer D., George L., Kit Tse C. (1990). Depression, disability days and days lost from work in a prospective epidemiological survey. JAMA.

[6] Social Security Superintendence (2019). National Statistics on Medical Licenses and Work Disability Subsidies. https://www.suseso.cl/608/w3-article-580746.html.

[7] Castro E. (2000). La Salud Mental del Profesor en Chile: Antecedentes para un Estado del Arte.

[8] Molina S. (2000). Logros de la década de los noventa y desafíos futuros. Rev. Perspectivas.

[9] CIDE Las Reformas Educativas: Logros, Problemas y Desafíos. https://www.cide.cl/cidehoja/_16_pdf.

[10] Schiefelbein E., Schiefelbein P. (2000). Determinantes de la calidad: ¿qué falta mejorar?. Rev. Perspectivas.

[11] UNESCO, OREALC, LLECE (2008). Los Aprendizajes de los Estudiantes de América Latina y el Caribe: Primer Reporte de los Resultados del Segundo Estudio Regional Comparativo y Explicativo (SERCE).

[12] LLECE Tercer Estudio Regional Comparativo y Explicativo (TERCE 2013). https://es.unesco.org/fieldoffice/santiago/llece/TERCE2013.

[13] Travers C., Cooper C. (1997). El Estrés de los Profesores: La Presión en la Actividad Docente.

[14] Golembiewski R., Munzenrider R., Carter D. (1983). Phases of progressive burnout and their work site covariant: Critical issues in OD research and praxis. Appl. Behav. Sci..

[15] Oramas A., Almirall P., Fernández I. (2007). Estrés Laboral y el Síndrome de Burnout en Docentes Venezolanos. Rev. Salud Trab..

[16] Fernández-Espejo H.A., Solari-Montenegro G.C. (2017). Prevalence of stress associated to the double presence and psychosocial factors in workers Chilean students. Cienc. Trab..

[17] Borgmann L.-S., Rattay P., Lampert T. (2020). Longitudinal Analysis of Work-to-Family Conflict and Self-Reported General Health among Working Parents in Germany. Int. J. Environ. Res. Public Health.

[18] Cerda J., Vera C., Rada G. (2013). Odds ratio: Theoretical and practical issues. Rev. Méd. Chile.

[19] Social Security Superintendence (2016). Monthly Overview Safety and Health at Work. Psychosocial Risk in Chile. Results of the Application of the SUSESO/ISTAS21 Questionnaire. https://www.suseso.cl/607/w3-article-18984.html.

[20] Social Security Superintendence (2017). Monthly Overview Safety and Health at Work. Psychosocial Risk in Chile. Results of the Application of the SUSESO/ISTAS21 Questionnaire. https://www.suseso.cl/607/w3-article-480616.html.

[21] Social Security Superintendence (2018). Monthly Overview Safety and Health at Work. Psychosocial Risk in Chile. Results of the Application of the SUSESO/ISTAS21 Questionnaire. https://www.suseso.cl/607/w3-article-577950.html.

[22] Social Security Superintendence (2019). Monthly Overview Safety and Health at Work. Psychosocial Risk in Chile. Results of the Application of the SUSESO/ISTAS21 Questionnaire. https://www.suseso.cl/607/w3-article-582169.html.

[23] Lodolo-D’Oria V., Pecori-Giraldi F., Della-Torre M., Tossa-Fasano A., Vizzi F., Fontani S., Vitello A., Cantoni S., Pascale A., Frigoli P. (2004). Is there any correlation between psychiatric disease and the teaching profession?. Med. Del. Lav..

[24] Tarafdar M., Gupta A., Turel O. (2013). The dark side of information technology use. Inf. Syst. J..

[25] Seong-Tak O., Park S. (2016). A Study of the Connected Smart Worker’s Techno-Stress. Proc. Comp. Sci..

[26] Gaudioso F., Turel O., Galimberti C. (2017). The mediating roles of strain facets and coping strategies in translating techno-stressors into adverse job outcomes. Comput. Hum. Behav..

[27] Day A., Paquet S., Scott N., Hambley L. (2012). Perceived Information and Communication Technology (ICT) Demands on Employee Outcomes: The Moderating Effect of Organizational ICT Support. J. Occup. Health Psychol..

[28] Sauter S.L., Murphy L.R., Hurrell J.J., Levi L., Stellman J.M. (1998). Psychosocial and Organizational Factors. Health and Safety at Work Encyclopedia.

[29] Salanova M., Llorens S., Cifre E., Nogareda C. (2007). The Technostress: Concept, Measurement, and Psychosocial Intervention. Prevention Technical Note 730, 21th Serie.

[30] Charria V.H., Sarsosa K.V., Arenas F. (2011). Occupational psychosocial risk factors: Methods and assessment tools. Rev. Fac. Nac. Salud Pública.

[31] Vega-Muñoz A., Estrada-Muñoz C., Realyvásquez-Vargas A., Arredondo-Soto K., Hernández-Escobedo G., González-Reséndiz J. (2020). Evaluating Technostress to Improve Teaching Performance: Chilean Higher Education Case. Evaluating Mental Workload for Improved Workplace Performance.

[32] Brod C. (1982). Managing Technostress-Optimizing the Use of Computer-Technology. Pers. J..

[33] Lee Y.K., Chang C.T., Lin Y., Cheng Z.H. (2014). The dark side of smartphone usage: Psychological traits, compulsive behavior and technostress. Comput. Hum. Behav..

[34] Riedl R., Kindermann H., Auinger A., Javor A. (2012). Technostress from a Neurobiological Perspective: System Breakdown Increases the Stress Hormone Cortisol in Computer Users. Bus. Inf. Syst. Eng..

[35] Wang K.L., Shu Q., Tu Q. (2008). Technostress under different organizational environments: An empirical investigation. Comput. Hum. Behav..

[36] Ragu-Nathan T.S., Tarafdar M., Ragu-Nathan B.S., Tu Q. (2008). The Consequences of Technostress for End Users in Organizations: Conceptual Development and Empirical Validation. Inf. Syst. Res..

[37] Turel O., Gaudioso F. (2018). Techno-stressors, distress and strain: The roles of leadership and competitive climates. Cogn. Technol. Work.

[38] Ayyagari R., Grover V., Purvis R. (2011). Technostress: Technological Antecedents and Implications. MIS Q..

[39] Cohen F., Lazarus R.S. (1973). Active Coping Processes, Coping Dispositions, and Recovery from Surgery. Psychosom. Med..

[40] Tarafdar M., Cooper C.L., Stich J.F. (2019). The technostress trifecta-techno eustress, techno distress and design: Theoretical directions and an agenda for research. Inf. Syst. J..

[41] Salanova-Soria M. (2003). Working with technologies and coping with technostress: The role of efficacy beliefs. Rev. Psicol. Trab. Organ..

[42] Lazarus R.S., Launier R., Pervin L.A., Lewis M. (1978). Stress-Related Transactions between Person and Environment. Perspectives in Interactional Psychology.

[43] Korukonda A.R. (2005). Personality, individual characteristics, and predisposition to technophobia: Some answers, questions, and points to ponder about. Inf. Sci..

[44] Choi S.B., Lim M.S. (2016). Effects of social and technology overload on psychological well-being in young South Korean adults: The mediatory role of social network service addiction. Comput. Hum. Behav..

[45] Rose P.M., Stoklosa K., Gray S.A. (1998). A focus group approach to assessing technostress at the reference desk. Ref. User Serv. Q..

[46] Poole C.E., Denny E. (2001). Technological change in the workplace: A statewide survey of community college library and learning resources personnel. Coll. Res. Libr..

[47] Hsiao K.L., Shu Y., Huang T.C. (2017). Exploring the effect of compulsive social app usage on technostress and academic performance: Perspectives from personality traits. Telemat. Inform..

[48] Cao X., Masood A., Luqman A., Ali A. (2018). Excessive use of mobile social networking sites and poor academic performance: Antecedents and consequences from stressor-strain-outcome perspective. Comput. Hum. Behav..

[49] Qi C. (2019). A double-edged sword? Exploring the impact of students’ academic usage of mobile devices on technostress and academic performance. Behav. Inf. Technol..

[50] Wang X., Tan S.C., Li L. (2020). Technostress in university students’ technology-enhanced learning: An investigation from multidimensional person-environment misfit. Comput. Hum. Behav..

[51] Yu T.K., Lin M.L., Liao Y.K. (2017). Understanding factors influencing information communication technology adoption behavior: The moderators of information literacy and digital skills. Comput. Hum. Behav..

[52] Verkijika S.F. (2019). Investigating teacher stress when using technology. Comput. Educ..

[53] Al-Fudail M., Mellar H. (2008). Investigating teacher stress when using technology. Comput. Educ..

[54] Burke M.S. (2009). The incidence of technological stress among baccalaureate nurse educators using technology during course preparation and delivery. Nurse Educ. Today.

[55] Jena R.K. (2015). Technostress in ICT enabled collaborative learning environment: An empirical study among Indian academician. Comput. Hum. Behav..

[56] Joo Y.J., Lim K.Y., Kim N.H. (2016). The effects of secondary teachers’ technostress on the intention to use technology in South Korea. Comput. Educ..

[57] Wang X., Li B. (2019). Technostress Among University Teachers in Higher Education: A Study Using Multidimensional Person-Environment Misfit Theory. Front. Psychol..

[58] Dong Y., Xu C., Chai C.S., Zhai X. (2020). Exploring the Structural Relationship Among Teachers’ Technostress, Technological Pedagogical Content Knowledge (TPACK), Computer Self-efficacy and School Support. Asia-Pacific Edu. Res..

[59] Li L., Wang X. (2020). Technostress inhibitors and creators and their impacts on university teachers’ work performance in higher education. Cogn. Technol. Work.

[60] Schmidt D.A., Baran E., Thompson A.D., Mishra P., Koehler M.J., Shin T.S. (2009). Technological Pedagogical Content Knowledge (TPACK). J. Res. Technol. Educ..

[61] Kish L. (1972). Muestreo de Encuesta.

[62] Sierra R. (2003). Técnicas de Investigación Social.

[63] Ministry of Education Open Data Mineduc. http://datosabiertos.mineduc.cl/docentes-asistentes-la-educacion/.

[64] Salanova M., Llorens S., Cifre E. (2013). The dark side of technologies: Technostress among users of information and communication technologies. Intern. J. Psychol..

[65] Kyriacou C., Sutcliffe J. (1978). Teacher Stress: Prevalence, Sources, and Symptoms. Brit. J. Educ. Psychol..

[66] Manso-Pinto J.F. (1989). Occupational Stress Factors as Perceived by Chilean School Teachers. J. Soc. Psychol..

[67] Mccormick J., Shi G. (1999). Teachers’ attributions of responsibility for their occupational stress in the People’s Republic of China and Australia. Brit. J. Educ. Psychol..

[68] Ferguson K., Mang C., Frost L. (2017). Teacher stress and social support usage. Brock Educ. J. Educ. Res. Pract..

[69] Boshoff S.M., Potgieter J.C., Ellis S.M., Mentz K., Malan L. (2018). Validation of the Teacher Stress Inventory (TSI) in a multicultural context: The SABPA study. S. Afr. J. Educ..

[70] Lozares-Colina C., López-Roldán P. (1991). El análisis de componentes principales: Aplicación al análisis de datos secundarios. Pap. Rev. Sociol..

[71] Boyle G.J., Borg M.G., Falzon J.M., Baglioni A.J. (1995). A structural model of the dimensions of teacher stress. Brit. J. Educ. Psychol..

[72] Wickham H. (2017). RStudio. Tidyverse: Easily Install and Load the «Tidyverse» (Versión 1.2.1) [Computer Software]. https://CRAN.R-project.org/package=tidyverse.

[73] Comtois D. (2019). Summarytools: Tools to Quickly and Neatly Summarize Data (Versión 0.9.3) [Computer Software]. https://CRAN.R-project.org/package=summarytools.

[74] Husson F., Josse J., Le S., Mazet J. (2019). FactoMineR: Multivariate Exploratory Data Analysis and Data Mining (Versión 1.42) [Computer Software]. https://CRAN.R-project.org/package=FactoMineR.

[75] Kassambara A., Mundt F. (2017). Factoextra: Extract and Visualize the Results of Multivariate Data Analyses (Versión 1.0.5) [Computer Software]. https://CRAN.R-project.org/package=factoextra.

[76] Maier M.J. (2015). REdaS: Companion Package to the Book R: Einführung durch angewandte Statistik (Versión 0.9.3) [Computer Software]. https://CRAN.R-project.org/package=REdaS.

[77] Revelle W. (2020). Psych.: Procedures for Psychological, Psychometric, and Personality Research (Versión 1.9.12.31) [Computer Software]. https://CRAN.R-project.org/package=psych.

[78] Rosseel Y. (2012). lavaan: An R Package for Structural Equation. Modeling. J. Statist. Soft.

[79] Hair J., Anderson R., Tatham R., Black W. (1998). Multivariate Data Analysis.

[80] Bentler P.M. (1990). Comparative Fit Indexes in Structural Models. Psychol. Bull..

[81] Browne M.W., Cudeck R., Bollen K.A., Long J.S. (1993). Alternative ways of assessing model fit. Testing Structural Equation Models.

[82] Tucker L.R., Lewis C. (1973). A reliability coefficient for maximum likelihood factor analysis. Psychometrika.

[83] Hu L., Bentler P.M. (1999). Cutoff criteria for fit indexes in covariance structure analysis: Conventional criteria versus new alternatives. Struct. Eq. Model..

[84] Heckhausen J., Schulz R. (1995). A life-span theory of control. Psychol. Rev..

[85] Salthouse T.A. (2004). What and when of cognitive aging. Curr. Direct. Psychol. Sci..

[86] Tams S., Davis F.D., Riedl R., vom Brocke J., Le’ger P.M., Randolph A.B. (2017). A Refined Examination of Worker Age and Stress: Explaining How, and Why, Older Workers Are Especially Techno-Stressed in the Interruption Age. Information Systems and Neuroscience: Gmunden Retreat on NeuroIS 2016.

[87] Scheibe S., Carstensen L.L. (2010). Emotional Aging: Recent Findings and Future Trends. J. Gerontol. Ser. B Psychol. Sci. Soc. Sci..

[88] Diehl M., Hay E.L. (2010). Risk and resilience factors in coping with daily stress in adulthood: The role of age, self-concept incoherence, and personal control. Dev. Psychol..

[89] Makhbul Z.M., Hasun F.M. (2011). Gender responses to stress outcomes. J. Glob. Manag..

[90] Bocchino C.C., Hartman B.W., Foley P.F. (2003). The relationship between person-organization congruence, perceived violations of the psychological contract, and occupational stress symptoms. Consult. Psychol. J. Pract. Res..

[91] Loosemore M., Waters T. (2004). Gender differences in occupational stress among professionals in the construction industry. J. Manag. Eng.

[92] Morash M., Kwak D.-H., Haarr R. (2006). Gender differences in the predictors of police stress. Policing.

[93] Antoniou A.S., Polychroni F., Viachakis A.N. (2006). Gender and age differences in occupational stress and professional burnout between primary and high-school teachers in Greece. J. Manag. Psychol..

[94] Fernandes C.F.V., Kumar S., Mekoth N. (2009). Gender differences in stress among bank officers of private public sectors. ICFAI J. Org. Behav..

[95] Hart J.L., Cress C.M. (2008). Are women faculty just “worrywarts?” Accounting for gender differences in self-reported stress. J. Hum. Behav. Soc. Environ..

[96] Liu C., Spector P.E., Shi L. (2008). Use of both qualitative and quantitative approaches to study job stress in different gender and occupational groups. J. Occu Health Psychol..

[97] de Smet P., Sans S., Dramaix M., Boulenguez C., de Backer G., Ferrario M., Kornitzer M. (2005). Gender and regional differences in perceived job stress across Europe. Eur. J. Public Health.

[98] Ergeneli A., Ilsev A., Karapınar P.B. (2010). Work-family conflict and job satisfaction relationship: The roles of gender and interpretive habits. Gend. Work. Org..

[99] Lambert E.G., Altheimer I., Hogan N.L. (2010). An exploratory examination of a gendered model of the effects of role stressors. Women Crim. Justice.

[100] Ministry of Labor and Social Welfare (2020). Law 21220 Modifies the Labor Code on Distance Work. http://bcn.cl/2dgpk.

[101] Ministry of Education (2019). Law 20903 Creates the Teacher Professional Development System and Modifies other Regulations. http://bcn.cl/1uzzn.

[102] Ministry of Education (2020). Decree with Force of Law 1 Fixes a Consolidated, Coordinated, and Systematized Text of Law Nº 19.070 that Approved the Statute of Education Professionals, and of the Laws that Complement and Modify It. http://bcn.cl/1uy48.

[103] Ministry of Education (2019). Decree with the Force of Law 2 Fixes a Consolidated, Coordinated, and Systematized Text of the Law nº20.370 with the Non-repealed Norms of the Decree with the Force of Law No. 1, of 2005. http://bcn.cl/1uxh9.

[104] Social Security Superintendence Work Licenses Issued by Medical Statistics, Common Origin Due to Mental Illness (Based on Electronic Work Licenses Issued by Medical). https://www.suseso.cl/607/articles-592232_archivo_01.pdf.

[105] Jalil-Milad R. (2019). Chilean springtime. ARS Med..

[106] Villalobos G.H. (2017). Methodological advances in the determination of the origin of stress-related occupational diseases: The Colombian experience. Rev. Bras. Med. Trab..

